# Ketogenesis impact on liver metabolism revealed by proteomics of lysine β-hydroxybutyrylation

**DOI:** 10.1016/j.celrep.2021.109487

**Published:** 2021-08-03

**Authors:** Kevin B. Koronowski, Carolina M. Greco, He Huang, Jin-Kwang Kim, Jennifer L. Fribourgh, Priya Crosby, Lavina Mathur, Xuelian Ren, Carrie L. Partch, Cholsoon Jang, Feng Qiao, Yingming Zhao, Paolo Sassone-Corsi

**Affiliations:** 1 Center for Epigenetics and Metabolism, U1233 INSERM, Department of Biological Chemistry, University of California, Irvine, Irvine, CA 92697, USA; 2 Ben May Department for Cancer Research, University of Chicago, Chicago, IL 60637, USA; 3 Shanghai Institute of Materia Medica, Chinese Academy of Sciences, Shanghai 201203, China; 4 Department of Biological Chemistry, University of California, Irvine School of Medicine, Irvine, CA 92697, USA; 5 Department of Chemistry and Biochemistry, University of California, Santa Cruz, Santa Cruz, CA 95064, USA; 6 Center for Circadian Biology, University of California, San Diego, La Jolla, CA 92093, USA; 7 These authors contributed equally; 8 Lead contact

## Abstract

Ketone bodies are bioactive metabolites that function as energy substrates, signaling molecules, and regulators of histone modifications. β-hydroxybutyrate (β-OHB) is utilized in lysine β-hydroxybutyrylation (Kbhb) of histones, and associates with starvation-responsive genes, effectively coupling ketogenic metabolism with gene expression. The emerging diversity of the lysine acylation landscape prompted us to investigate the full proteomic impact of Kbhb. Global protein Kbhb is induced in a tissue-specific manner by a variety of interventions that evoke β-OHB. Mass spectrometry analysis of the β-hydroxybutyrylome in mouse liver revealed 891 sites of Kbhb within 267 proteins enriched for fatty acid, amino acid, detoxification, and one-carbon metabolic pathways. Kbhb inhibits S-adenosyl-L-homocysteine hydrolase (AHCY), a rate-limiting enzyme of the methionine cycle, in parallel with altered metabolite levels. Our results illuminate the role of Kbhb in hepatic metabolism under ketogenic conditions and demonstrate a functional consequence of this modification on a central metabolic enzyme.

## In brief

Lysine acylations are diverse and often metabolism-linked posttranslational modifications. Koronowski et al. find that dietary and disease states of ketogenesis evoke lysine β-hydroxybutyrylation (Kbhb) across the cellular proteome. In liver, a functional consequence of Kbhb is inhibition of the rate-limiting methionine cycle enzyme AHCY.

## Graphical Abstract

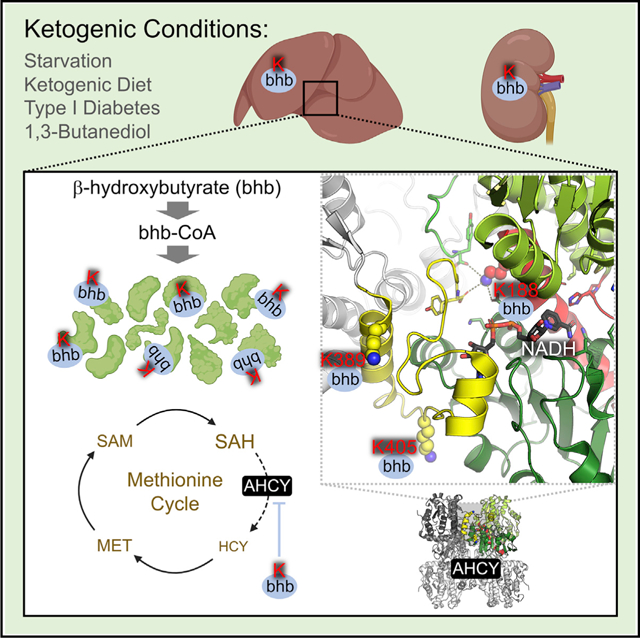

## INTRODUCTION

Ketogenesis provides metabolic intermediates and alternative fuels in many mammalian species (Puchalska and Crawford, 2017; Cahill, 2006). In the liver, fatty acid oxidation-derived acetyl-CoA produces β-hydroxybutyrate (β-OHB), acetoacetate, and acetone, three primary ketone bodies that are circulated among extrahepatic tissues and metabolized. This process is leveraged during periods of high fatty acid oxidation and diminished carbohydrate availability, as is evident during the neonatal period, fasting, starvation, prolonged exercise, and adherence to a ketogenic diet (KD), where it can become a major contributor to whole-organismal metabolism (Koeslag et al., 1980; McGarry and Foster, 1980; Balasse and Féry, 1989). Physiological ketone body concentration in the blood ranges from 100 to 250 μM, although levels increase to ~1 mM with a 24-h fast and can reach ~20 mM in pathological states such as uncontrolled diabetes (Balasse and Féry, 1989; Cahill, 2006). The most abundant ketone body, β-OHB, is particularly intriguing given its diverse bioactive properties. Although the predominant fate of β-OHB is terminal oxidation as an energy substrate, evidence demonstrates its involvement in cellular signaling and posttranslational modification (PTM) of histone lysines (Puchalska and Crawford, 2017; Xie et al., 2016; Ruan and Crawford, 2018).

Lysine acetylation (Kac) is directly linked to cellular metabolism (Peleg et al., 2016; Katada et al., 2012). Levels of acetyl-CoA, along with acetyltransferase and deacetylase enzymes, dynamically regulate protein acetylation in response to changes in metabolic flux through corresponding pathways, for example, the TCA cycle and fatty acid oxidation (Choudhary et al., 2009; Verdin and Ott, 2015). Other acyl-CoA metabolites, many of which are key intermediates in cellular metabolism, support a diversity of acyllysine modifications, including succinylation, malonylation, glutarylation, crotonylation, and others (Wagner and Hirschey, 2014; Ringel et al., 2018; Sabari et al., 2015; Rardin et al., 2013a). As alluded to, β-OHB serves as a substrate for histone lysine β-hydroxybutyrylation (Kbhb) via its activated thioester form β-hydroxybutyryl-CoA (Xie et al., 2016). In starved mice, increased β-OHB levels result in Kbhb on many histone lysine residues in the liver. Specifically, H3K9bhb associates with a set of starvation-responsive genes that are distinct from genes that associate with acetylation at the same H3K9 residue (Xie et al., 2016). Thus, Kbhb facilitates the adaptive response to starvation by coupling metabolism to gene expression.

Lysine acylation occurs on a large variety of non-histone proteins, adding another layer of regulation to diverse cellular and metabolic pathways (Carrico et al., 2018; Lin et al., 2012). Characterization of the proteome-wide lysine β-hydroxybutyrylome is an essential step to define Kbhb cellular targets and enable investigation of the impact of ketogenesisatthe cellular level. Here, weshow the induction of global protein Kbhb in the liver and kidney under various conditions of elevated β-OHB. We identified 891 sites of Kbhb within 267 proteins in starved liver. These belong to macronutrient, detoxification, and one-carbon metabolic pathways, among others. Furthermore, we demonstrate that Kbhb directly inhibits the activity of the rate-limiting methionine cycle enzyme S-adenosyl-L-homocysteine hydrolase (AHCY), concurrent with alterations of methionine cycle metabolites. Our results reveal the full landscape of Kbhb and demonstrate a regulatory mechanism of Kbhb on a key metabolic enzyme under ketogenesis, implicating Kbhb in physiological and pathological processes.

## RESULTS

### Global protein β-hydroxybutyrylation *in vivo* and *in vitro*

**Figure 1.**
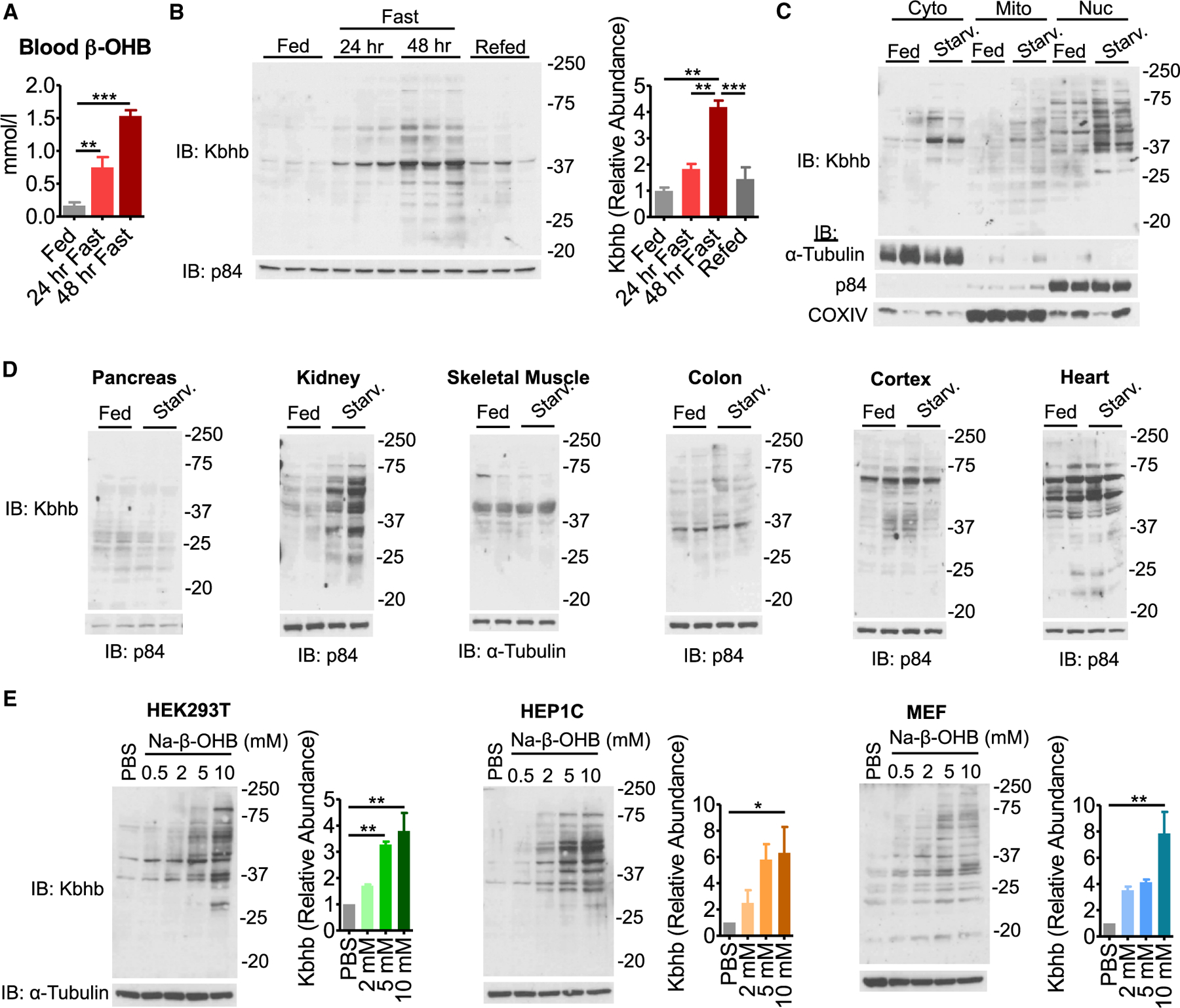
β-hydroxybutyrate is linked to global protein Kbhb *in vivo* and *in vitro* (A) Blood β-hydroxybutyrate (β-OHB) levels in fed versus starved (48-h fast) mice. n = 3–6, unpaired Student’s t test, ***p < 0.001. (B) Western blot for pan-β-hydroxybutyryllysine (Kbhb) from liver whole-cell lysates. n = 3 replicates are quantified (right). Unpaired Student’s t test, **p < 0.01. (C) Western blot from livers fractionated into cytosolic (Cyto), mitochondrial (Mito), and nuclear (Nuc) compartments. Fraction enrichment is demonstrated by compartment-specific loading controls. (D) Whole-cell lysates were prepared from metabolic tissues to probe Kbhb by western blot. (E) Representative western blots from cultured cell lines treated with dose-response amounts of sodium-β-hydroxybutyrate (Na-β-OHB) for 24 h. n = 3 replicates are quantified (right). One-way ANOVA, *p < 0.05, **p < 0.01. Data are represented as mean ± SEM. See also Figure S1.

To assess the extent of Kbhb on cellular metabolism, a pan-β-hydroxybutyryllysine antibody was used to probe a panel of mouse tissues under various physiological conditions. As reported previously, starvation (48-h fasting) elevated blood β-OHB and increased histone Kbhb in the liver (Figures 1A and S1A). Remarkably, whole-cell lysates showed a similarly significant induction of Kbhb signal for proteins of various molecular weights (Figure 1B; Xie et al., 2016). Probing subcellular fractions revealed that Kbhb was induced broadly on cytosolic, mitochondrial, and nuclear proteins (Figure 1C). Because β-OHB is released from the liver and metabolized throughout the body, we probed other metabolic tissues. Starvation markedly induced Kbhb in the kidney but not pancreas, skeletal muscle, colon, heart, or cerebral cortex (Figure 1D), suggesting that tissue-specific metabolism of β-OHB influences Kbhb. To assess the extent of protein Kbhb *in vitro*, cultured cells were treated with sodium-β-OHB (Na-β-OHB) for 24 h. Mouse HEP1C, mouse embryonic fibroblast (MEF), and human HEK293T cells displayed concentration-dependent increases in protein Kbhb in response to treatment (Figure 1E). Together, these data demonstrate global protein Kbhb.

### Ketogenic conditions and β-OHB induce protein β-hydroxybutyrylation

**Figure 2.**
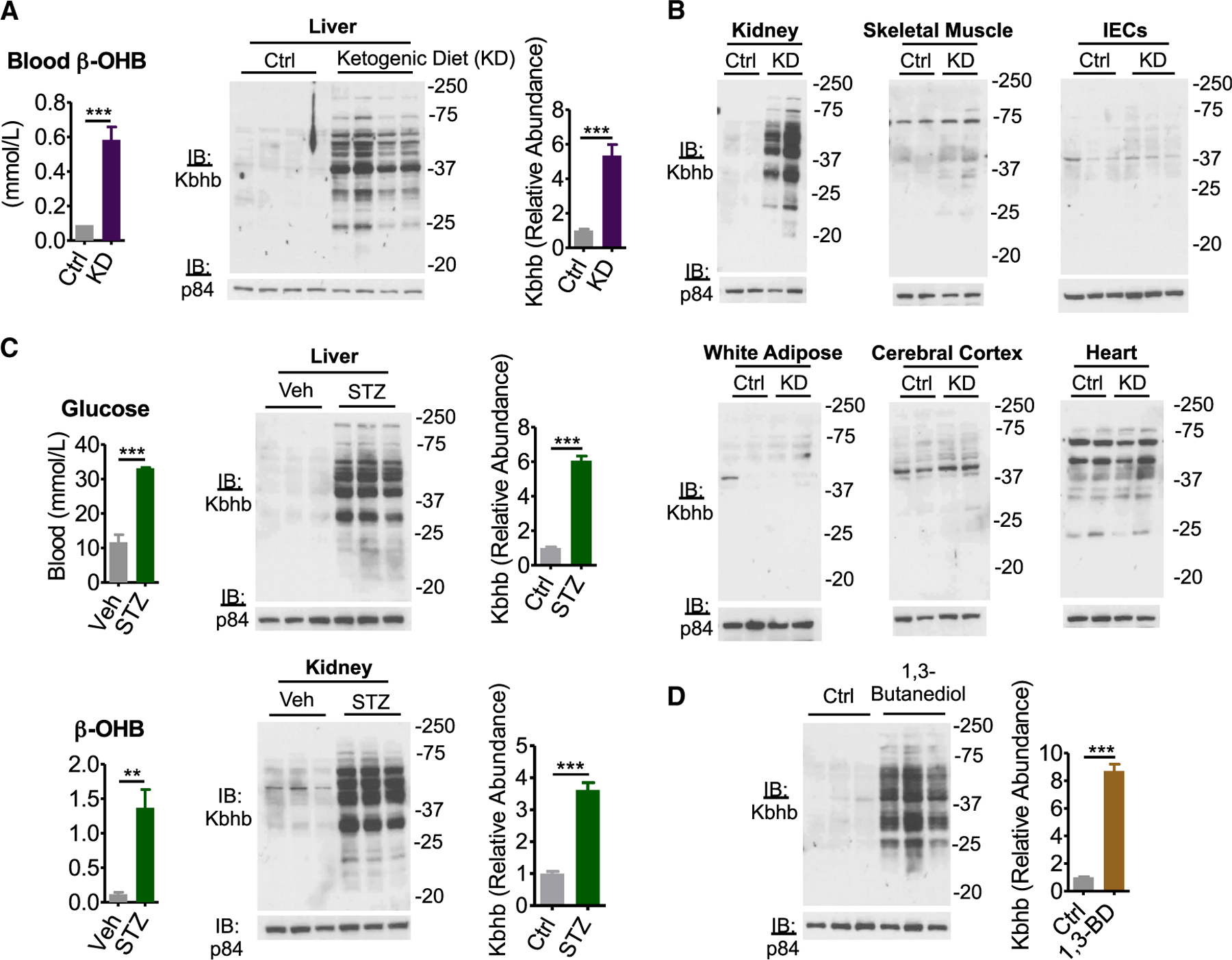
Various ketogenic conditions present with global protein β-hydroxybutyrylation (A) Left: blood β-OHB levels in mice fed control (Ctrl) or ketogenic diet (KD) for 4 weeks. n = 6, unpaired Student’s t test, ***p < 0.001. Right: western blot for pan-β-hydroxybutyryllysine (Kbhb) from liver whole-cell lysates. n = 4 replicates are quantified (right), unpaired Student’s t test, ***p < 0.001. (B) Whole-cell lysates from Ctrl or KD conditions of the indicated tissue. IECs, intestinal epithelial cells. (C) Type I diabetes mellitus was induced by intraperitoneal injection of vehicle (Veh) or 200 mg/kg streptozotocin (STZ). Blood measurements were taken (left) and tissue whole-cell lysates (right) were prepared 4 d post injection. n = 3 replicates for liver are quantified. Unpaired Student’s t test, **p < 0.01, ***p < 0.01. (D) Liver whole-cell lysates from mice fed Ctrl or 10% (w/w) 1,3-butanediol (1,3-BD) diet were probed by western blot. n = 3 replicates are quantified, unpaired Student’s t test, ***p < 0.001. Data are represented as mean ± SEM. See also Figure S2.

Assuming that Kbhb is a general consequence of ketogenesis, then various conditions that evoke elevation of β-OHB production should also evoke Kbhb. We placed mice on a KD for 4 weeks, which elicited ketogenesis and increased β-OHB (Figure 2A; Newman et al., 2017; Sato et al., 2017; Tognini et al., 2017). KD stimulated global protein Kbhb in the liver and kidney, but not other metabolic tissues, as observed under starvation (Figures 2A and 2B). Because KD induces circadian oscillations of β-OHB (Smith and Robinson, 2016), we collected samples along the circadian cycle and observed no apparent changes in Kbhb levels in western analyses for the proteins detected under these experimental conditions (Figure S2A).

Uncontrolled type I diabetes mellitus (TIDM) is a disease state that induces ketogenesis and presents with ketoacidosis (Ruan and Crawford, 2018). Mice were administered streptozotocin (STZ), which induces TIDM by β cell destruction (Furman, 2015), evidenced by hyperglycemia coinciding with an elevation of blood β-OHB (Figure 2C). Liver and kidney from STZ-treated mice displayed a marked increase of global protein Kbhb compared to the control treatment (Figure 2C).

To determine whether β-OHB alone is sufficient for Kbhb, we turned to 1,3-butanediol. This ketone precursor is converted to β-OHB by the hepatic alcohol dehydrogenase system and can be administered orally to increase β-OHB (Hashim and VanItallie, 2014; Veech, 2014). Livers from mice fed for 3 weeks a diet of 10% (w/w) 1,3-butanediol displayed a marked induction of protein Kbhb (Figure 2D). Together, these data demonstrate that dietary interventions, disease states, and β-OHB precursors evoke global protein Kbhb.

### Characterizing the lysine β-hydroxybutyrylome in mouse liver

**Figure 3.**
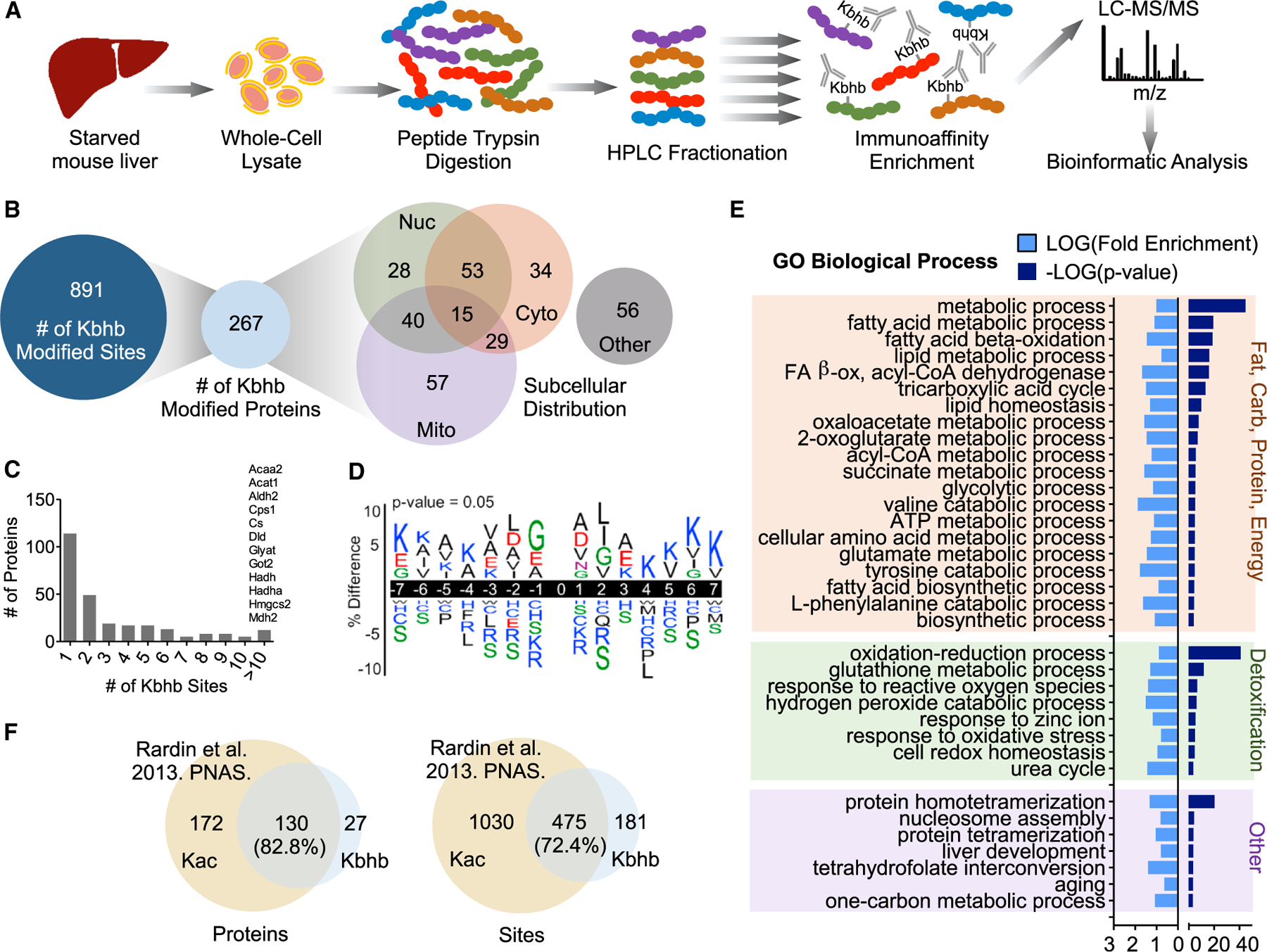
Characterizing the lysine β-hydroxybutyrylome in mouse liver (A) Schematic of β-hydroxybutyryllysine (Kbhb)-modified peptide identification. LC, liquid chromatography; MS, mass spectrometry. (B) The number of Kbhb sites and proteins and their subcellular distribution as determined by the COMPARTMENTS database. (C) Histogram of the number of Kbhb sites per protein. Proteins with more than 10 sites of Kbhb are listed above the corresponding bar. (D) Sequence logo of Kbhb sites determined by iceLogo. Amino acids are colored by side-chain properties: blue, positively charged; red, negatively charged; green, polar uncharged; black, hydrophobic. 0, modification site. (E) Results from DAVID Gene Ontology (GO) analysis. Significantly enriched pathways (p < 0.001) were categorized by cellular function for visualization. (F) Overlap of Kbhb proteins and sites with Kac proteins and sites from Rardin et al. (2013b) (24-h starved mouse liver mitochondrial fractions). Only mitochondrial proteins (determined by MitoMiner) were compared. See also Table S1.

We sought to identify the specific proteins and sites of Kbhb *in vivo*. To do so, we enriched Kbhb-modified peptides from starved mouse liver for analysis by mass spectrometry (MS) (Figure 3A). Proteins from whole-cell lysates were trypsin digested and the resulting peptides were fractionated by high-performance liquid chromatography (HPLC) to increase coverage depth. β-hydroxybutyrylated peptides were then isolated by immunoaffinity enrichment using a polyclonal pan-β-hydroxybutyryllysine antibody (Xie et al., 2016). Samples were analyzed by liquid chromatography-tandem mass spectrometry (LC-MS/MS) on an Orbitrap Velos mass spectrometer and data were mapped to the mouse proteome. Using this method, we identified 891 sites of Kbhb across 267 proteins (Figure 3B; Table S1). Examining their subcellular distribution revealed a widespread cellular impact, as all major compartments were represented (Figure 3B).

The number of sites per protein varied, with the majority (114,42.7%) having one site (Figure 3C). However, several key metabolic enzymes were heavily β-hydroxybutyrylated at more than 10 sites. For example, carbamoyl phosphate 1 (CPS1), a rate-limiting enzyme of the urea cycle, and HMGCS2, the rate-limiting enzyme of ketogenesis, contained 35 and 15 sites of Kbhb, respectively. To determine a possible consensus motif for Kbhb, we compared the amino acid sequences surrounding the modification sites using iceLogo (Colaert et al., 2009). Positions close to the modified site (−2 to +2) were modestly enriched with glycine (small and flexible) and hydrophobic side-chain-containing amino acids (leucine, alanine, and isoleucine) whereas the positively charged amino acids lysine and arginine, as well as serine (polar uncharged side chain), were excluded (Figure 3D).

To understand the potential regulation by Kbhb of liver function, we performed Gene Ontology (GO) analysis using DAVID (Huang et al., 2009). Numerous metabolic networks central to the liver were enriched with Kbhb-modified proteins; nevertheless, energy and detoxification pathways were the most apparent (Figure 3E). Nearly all macronutrient pathways were top hits, encompassing fatty acid (lipid and acyl-CoA metabolic processes, β-oxidation), TCA cycle, glycolytic/gluconeogenic, amino acid (several), ketone body (3 of 4 enzymes), and ATP metabolic processes. During prolonged fasting, several of these pathways are metabolizing ketogenic substrates such as fatty-acid-derived acetyl-CoA and ketogenic amino acids into ketone bodies (Puchalska and Crawford, 2017), and thus Kbhb appears to feed back onto the biosynthetic processes that induce it. Cellular detoxification pathways were also enriched, specifically glutathione metabolism, response to reactive oxygen species and oxidative stress, hydrogen peroxide catabolism, and redox homeostasis. Other notable enrichment included nucleosome assembly, evidenced by identification of Kbhb on various histone species, as well as cofactor pathways such as tetrahydrofolate interconversion and the one-carbon metabolic process. Notably, the one-carbon cycle and methionine cycle constitute a key metabolic node in the liver that links diverse metabolic pathways (Walker, 2017).

Kac is also induced under ketogenic conditions, as a substantial rise in acetyl-CoA feeds the production of β-OHB (Roberts et al., 2017; Sato et al., 2017). Thus, Kac occurs in parallel with Kbhb, as evident in liver whole-cell lysates from starved mice (Figure S3A). An important distinction to make is whether Kac and Kbhb target similar or distinct groups of proteins under these conditions. We thereby compared our Kbhb dataset with the previously determined lysine acetylome from 24-h fasted mouse liver (Rardin et al., 2013b). Because this dataset was generated from mitochondrial fractions, we limited our analysis to mitochondrial localized proteins as determined by MitoMiner (Smith and Robinson, 2016). This analysis revealed that 82.8% of Kbhb-modified proteins and 72.4% of Kbhb-modified sites are also targeted by Kac (Figure 3F), indicating significant overlap of the pathways targeted by these two modifications.

### β-OHB regulates AHCY activity

**Figure 4.**
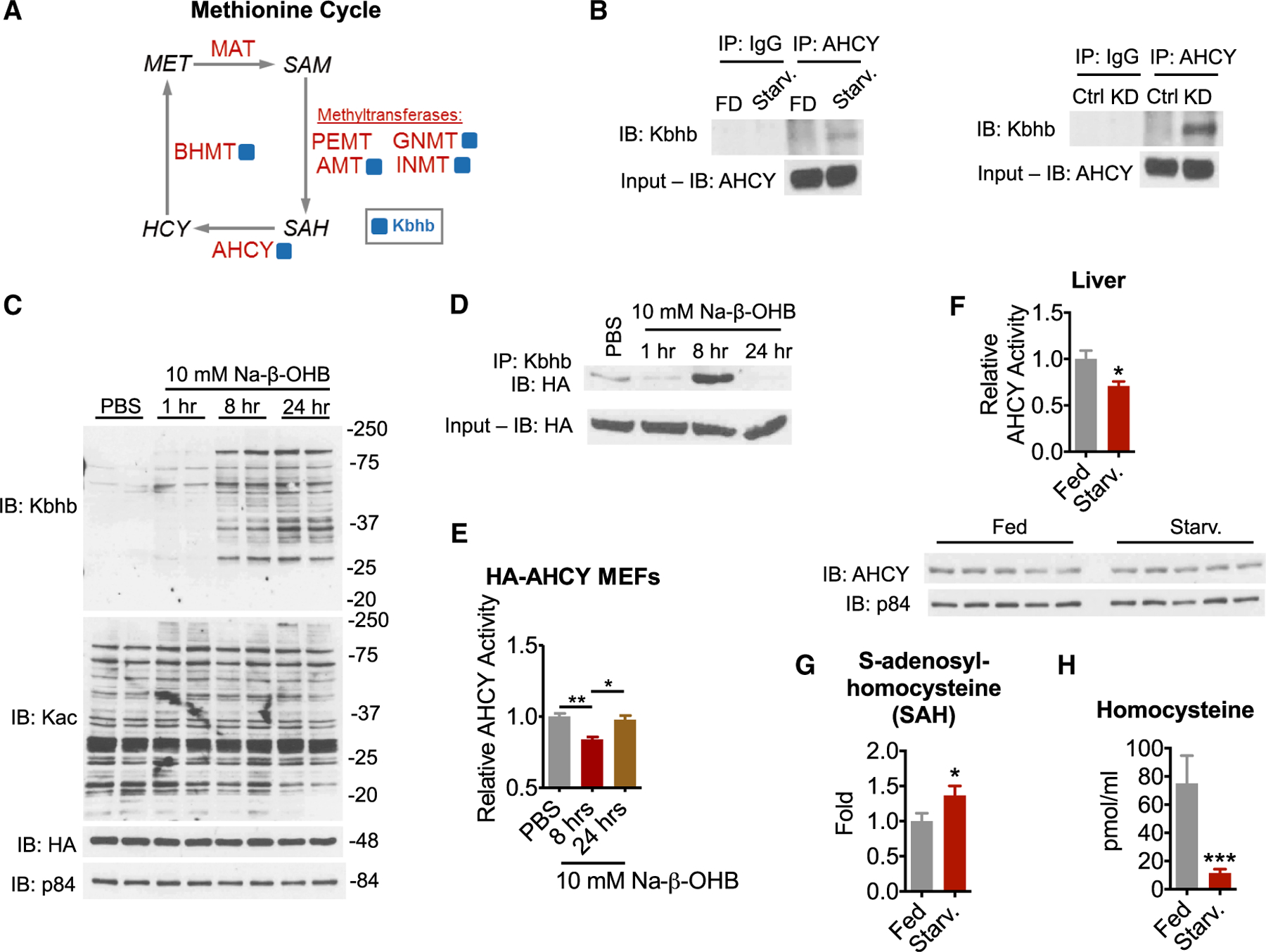
β-hydroxybutyrate inhibits AHCY enzymatic activity (A) Simplified schematic of the methionine cycle. Enzymes are in red, metabolites are in black, and blue squares indicate identification of Kbhb sites. MAT, S-adenosylmethionine synthetase; BHMT, betaine-homocysteine methyltransferase; AHCY, S-adenosyl-L-homocysteine hydrolase; PEMT, phosphatidylethanolamine *N*-methyltransferase; GNMT, glycine *N*-methyltransferase; INMT, indolethylamine *N*-methyltransferase; AMT, aminomethyltransferase; MET, methionine; SAM, S-adenosylmethionine; SAH, S-adenosylhomocysteine; HCY, homocysteine. (B) Immunoprecipitation (IP) of AHCY from liver whole-cell lysates. FD, fed; Starv., 48-h fast. (C) Time course of Kbhb induction in HA-AHCY MEF cells. (D) Time course of Kbhb of AHCY in HA-AHCY MEF cells revealed by immunoprecipitation. (E) AHCY activity was measured as the rate of adenosine production from SAH hydrolysis in HA-AHCY MEF whole-cell lysates. One-way ANOVA, *p < 0.05, **p < 0.01, n = 3. (F) AHCY activity was measured from liver whole-cell lysates as in (E). Unpaired Student’s t test, *p < 0.05, **p < 0.01, n = 8. AHCY protein levels from liver whole-cell lysates are shown below. (G) Relative S-adenosylhomocysteine levels in liver measured by LC-MS. Unpaired Student’s t test, *p < 0.05, n = 8–9. (H) Homocysteine concentration in serum. Unpaired Student’s t test, ***p < 0.001, n = 5–6. Data are represented as mean ± SEM. See also Figures S3 and S4.

One-carbon metabolism was among the top metabolic pathways enriched for Kbhb (Figure 3E). Notably, several enzymes implicated in methionine metabolism were identified by our MS analysis (Figure 4A). The importance of methionine metabolism for numerous cellular functions, ranging from biosynthesis of lipids and metabolites to epigenetic regulation through methylation of DNA, RNA, and histones (Serefidou et al., 2019; Haws et al., 2020; Ducker and Rabinowitz, 2017), prompted us to determine the effects of Kbhb on this pathway. We focused on AHCY, a rate-limiting enzyme that hydrolyzes S-adenosylhomocysteine (SAH) to homocysteine and adenosine (Figure 4A). Complete loss of AHCY leads to embryonic lethality (Dickinson et al., 2016), whereas AHCY deficiency in humans results in a number of pathological consequences including neurodevelopmental disorders, myopathy, hepatocellular carcinoma, and early childhood death (Barić, 2009; Stender et al., 2015).

Despite the biological importance of AHCY, little is known about its regulation. Thus, we sought to validate AHCY as a target of Kbhb by immunoprecipitation. Western blots showed that both starvation and KD induce Kbhb on AHCY compared to control conditions (Figure 4B). Neither starvation nor KD induced Kac on AHCY (Figure S4C). To assess the relative stoichiometry of Kbhb on AHCY, we immunoprecipitated peptides from KD livers with immunoglobulin G (IgG) or Kbhb antibodies. Immunoprecipitation with Kbhb depleted 24.67% of AHCY, demonstrating relatively high stoichiometry (Figure S4D) and plausible impact on activity of a rate-limiting enzyme. To further confirm Kbhb of AHCY, we stably expressed hemagglutinin (HA)-tagged AHCY in MEF cells. Comparable to wild-type (WT) MEF cells, 10 mM Na-β-OHB treatment induced global protein Kbhb in a time-dependent manner in HA-AHCY MEF cells (Figure 4C). Importantly, and in contrast to liver, Na-β-OHB treatment did not induce global protein acetylation (Figure 4C). Immunoprecipitation of HA-AHCY revealed a robust Kbhb signal for AHCY 8 h after treatment that was attenuated by 24 h (Figure 4D). These data show that AHCY is a bona fide target of Kbhb.

AHCY catalyzes the hydrolysis of SAH to homocysteine, freeing adenosine in the process. To determine whether Kbhb of AHCY coincides with a change in its enzymatic activity, we measured the rate of adenosine production from SAH hydrolysis in HA-AHCY MEF cells. Although protein levels remained constant (Figure 4C), AHCY activity was inhibited at 8 h following 10 mM Na-β-OHB treatment and returned to baseline by 24 h, mirroring the Kbhb signal (Figure 4E). Similar timing of AHCY inhibition in response to 10 mM Na-β-OHB was observed in WT MEF and HEK293T cells (Figures S4A and S4B). In starved liver, AHCY activity was attenuated whereas protein levels were unaltered (Figure 4F). To determine whether inhibition of AHCY coincided with alterations of methionine cycle metabolites, we measured levels of its substrate SAH and product homocysteine. SAH was increased in starved liver (Figure 4G). Homocysteine was markedly decreased in serum (Figure 4H). Together, these results indicate that β-OHB (and likely Kbhb) inhibits AHCY activity, concurrent with altered methionine cycle function.

### Kbhb of lysines within the NAD^+^-binding interface of AHCY inhibits its activity

**Figure 5.**
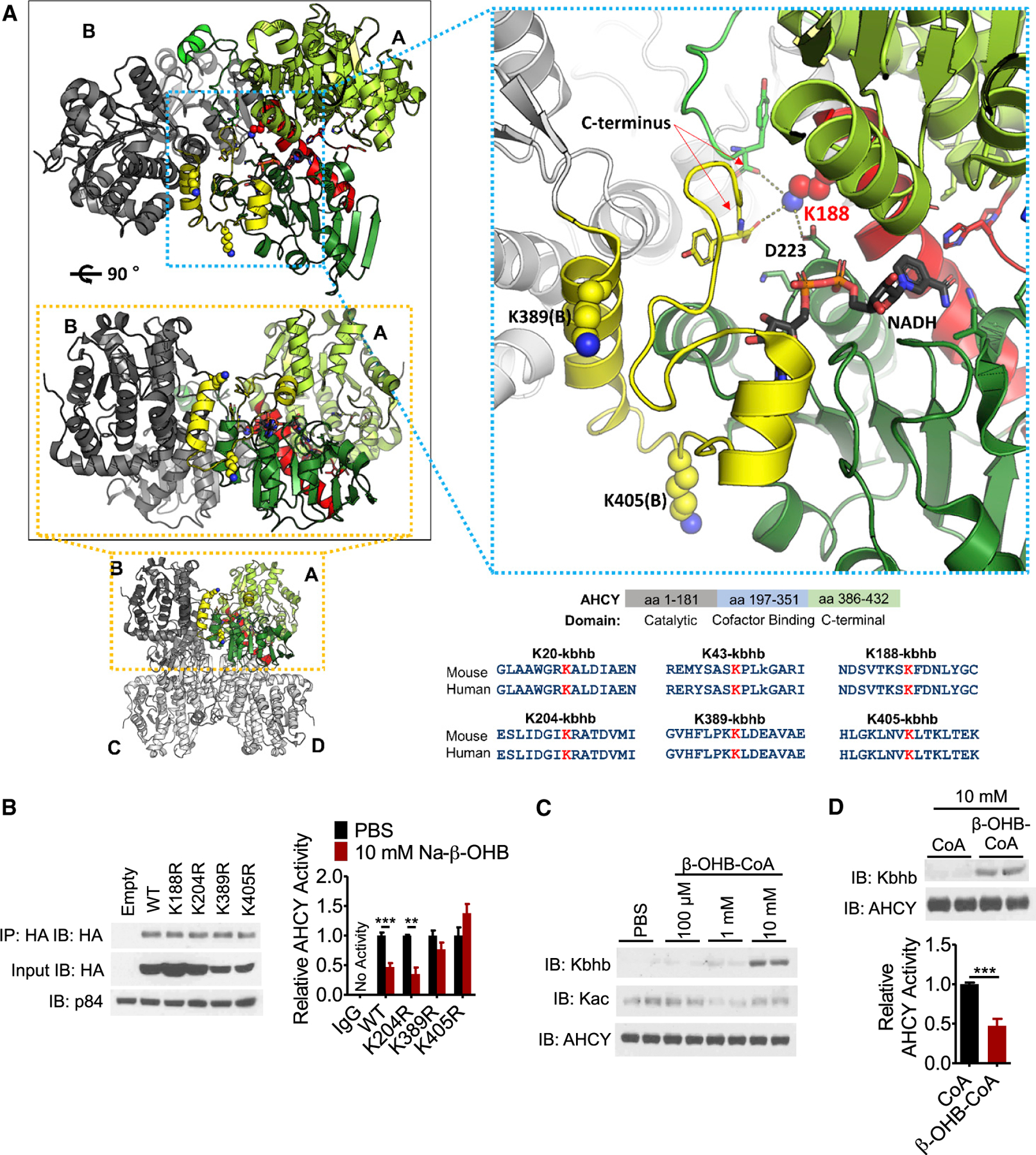
β-hydroxybutyrylation of K389 and K405 inhibits AHCY enzymatic activity (A) Crystal structure of AHCY (PDB: 4YVF) bound to NAD^+^/NADH. Four identical monomers form 2 dimers (AB and CD) and, in turn, 1 tetramer. The substrate-binding domain (light green) and cofactor-binding domain (dark green) of A, and the C-terminal loop of B (yellow), constitute one active site. The connecting helix and loop between the substrate-binding and cofactor-binding domains are shown in red. Kbhb sites are blue spheres (K188, K389[B], and K405[B]). The stick model of NAD^+^/NADH has black carbons. Dashed lines mark residues within hydrogen-bond distance from K188. Structures were generated with PyMOL. Bottom right: conservation of AHCY Kbhb sites from mouse to human. (B) HEK293T cells were transfected with WT or the indicated lysine mutant plasmid, treated for 10 h with 10 mM Na-β-OHB to induce Kbhb, and then transfected AHCY was immunoprecipitated by its HA tag and assayed for enzymatic activity; values are normalized to PBS for each plasmid (see also Figure S5B). Unpaired Student’s t test, **p < 0.01, ***p < 0.001; WT, n = 6; mutants, n = 3. (C) Recombinant AHCY protein was incubated with increasing concentrations of β-hydroxybutyryl-CoA (β-OHB-CoA) for 2 h at 37°C, pH 8.0. (D) AHCY activity was measured from recombinant protein incubated with CoA or β-OHB-CoA as in (C). Unpaired Student’s t test, ***p < 0.001; n = 4. Data are represented as mean ± SEM. See also Figure S5.

Considering Kbhb of AHCY parallels inhibition of its enzymatic activity, we sought to gain insight into how Kbhb may elicit this regulation. To do so, we examined the crystal structure of AHCY in its NAD^+^-bound form (Figure 5A). AHCY functions as a tetramer, composed of two homodimers of structurally identical ~48-kDa subunits that each contain a substrate-binding domain, cofactor-binding domain, and C-terminal loop (Grbeša et al., 2017). NAD^+^ binding to AHCY depends on the interaction between C-terminal loops of neighboring subunits, which together constitute one cofactor binding and substrate binding site (Wang et al., 2014). Four of the 6 Kbhb sites identified by our proteomic analysis are in close proximity to this critical interface (K188, K204, K389, and K405) whereas 2 are located on the surface, quite distal from the active site (K20 and K43). Our *in silico* analysis shows that K188 is functionally important for forming hydrogen-bond interactions with the C terminus of each homo-dimer and D223. K389 also participates in hydrogen-bond interactions between S420 and D422 on the C-terminal loops, whereas the amine nitrogen of K405 interacts with the carboxyl group of E264. These interactions seemingly help support the NAD^+^ binding site conformation and, in turn, the nearby substrate binding site. Kbhb of these lysine residues would introduce a positive charge, predictive of disruption of these interactions and stability of this interface. Ultimately, an unfavorable conformational change could impair enzymatic activity.

To test our hypothesis, we mutated candidate lysine residues to arginine (K→R), a structurally similar amino acid with the same charge, to mimic an unmodified lysine that cannot be subsequently β-hydroxybutyrylated. HEK293T cells, whose response to 10 mM Na-β-OHB treatment is comparable to HAMEF cells (Figure S5A), were transfected with WT, K188R, K204R, K389R, or K405R HA-AHCY plasmids and treated with 10 mM Na-β-OHB for 10 h to induce Kbhb. To avoid influence from endogenous AHCY, we immunoprecipitated the transfected AHCY by its HA tag and directly measured enzymatic activity. All constructs were expressed and immunoprecipitated at comparable levels (Figure 5B). Of the four mutations, K188R had a substantial effect on its own, almost completely blocking AHCY activity, demonstrating that K188 mediates a vital structural and functional interaction (Figure S5B). Next, we analyzed the contribution of each residue under Kbhb. Compared to PBS treatment, 10 mM Na-β-OHB inhibited WT AHCY activity (Figure 5B). The K204R mutant was inhibited by β-OHB equal to WT, demonstrating a negligible effect of Kbhb at this site (Figures 5B and S5B). In contrast, K389R and K405R abolished β-OHB-induced inhibition of AHCY activity (Figures 5B and S5B). Considering the position of these residues and the crystal structure modeling, we posit that Kbhb disrupts the critical C-terminal loop interactions involving K389 and K405, destabilizing the NAD^+^ binding site conformation and impairing enzymatic activity.

A synergistic effect of Kbhb at several sites including K389, K405, and K188 is likely, ultimately acting together in altering AHCY activity. Thus, we next determined the direct effect of β-hydroxybutyrylation on AHCY activity in a cell-free assay. Recombinant AHCY protein was incubated with β-hydroxybutyryl-CoA for 2 h at 3700B3ΰC and formation of Kbhb was monitored by western blot. β-hydroxybutyryl-CoA induced Kbhb on AHCY in a concentration-dependent manner, in the absence of any potential acylating enzyme (Figure 5C). This incubation did not cause Kac (Figure 5C). Compared to incubation with CoA, β-hydroxybutyryl-CoA reduced AHCY enzymatic activity (Figure 5D), similar to the effect of β-OHB observed in cells. These data show that Kbhb directly inhibits AHCY activity.

## DISCUSSION

**Figure 6.**
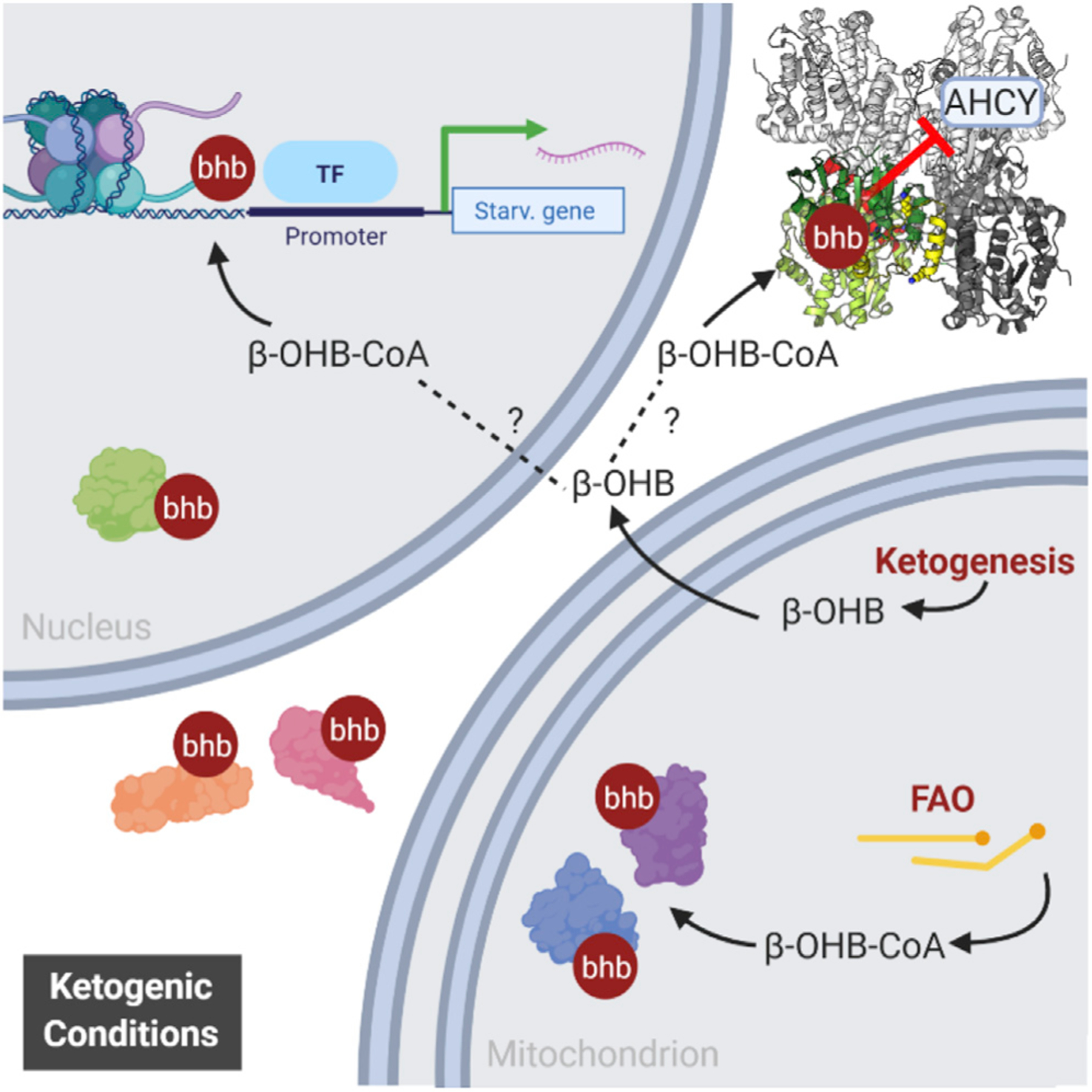
Model of Kbhb under ketogenic conditions In ketogenic liver, as the concentration of β-OHB rises, so does the concentration of its activated CoA form β-OHB-CoA, which serves as the substrate for Kbhb. β-OHB-CoA may be generated by mitochondrial enzymes that participate in fatty acid β-oxidation or by ACSS2, a nucleo-cytoplasmic enzyme that generates other short-chain acyl-CoA species. Kbhb has the potential to feed back on the hepatic proteome and impact metabolism—as demonstrated for AHCY and the methionine cycle—as well as alter gene expression through modification of histones.

In mice and humans, β-OHB elicits pleiotropic effects in numerous physiological and pathological contexts, often with potentially therapeutic value. Such conditions include prolonged physical exercise, caloric restriction, KD, uncontrolled diabetes, heart failure, neuroprotection, and certain cancers, among others (Puchalska and Crawford, 2017; Dąbek et al., 2020; Newman and Verdin, 2017). Here we report a proteome-level role for β-OHB in the regulation of hepatic metabolism. Using high-throughput proteomic analysis of Kbhb in liver, we reveal Kbhb as a widespread posttranslational modification of non-histone proteins (Figure 6). Focusing on the methionine cycle enzyme AHCY, we demonstrate the ability of Kbhb to alter the enzymatic activity of a key metabolic regulator. Finally, we implicate global protein Kbhb broadly in biological contexts by illuminating its presence in dietary, disease, and potentially therapeutic interventions.

Interestingly, the induction of global protein Kbhb appears highly tissue specific to the liver and kidney. During ketogenesis, the liver produces most, if not all, β-OHB and its own concentration parallels that of the blood (Puchalska and Crawford, 2017; Tognini et al., 2017). Reabsorption of β-OHB by the kidney is proportionate to blood levels, and kidney β-OHB levels are markedly increased by a 24-h fast (Puchalska and Crawford, 2017). Under these conditions the metabolic tasks of the liver and kidney are similar in that both organs help maintain circulating glucose by gluconeogenesis and provide ketone bodies through ketogenesis (although renal ketogenesis is debated) (Puchalska and Crawford, 2017). While hepatic and renal β-OHB levels rise, it flows down its concentration gradient through solute carriers 16A 1 and 7 (MCT1 and MCT2) into extrahepatic tissues, where it is rapidly metabolized to acetyl-CoA for terminal oxidation via the TCA cycle. Because heavy oxidizers of β-OHB such as the heart, skeletal muscle, and brain do not display Kbhb induction (Figures 1 and 2), it appears that Kbhb is tied to β-OHB levels in tissue. This notion keeps with the dose-dependent induction of Kbhb *in vitro* and *in vivo* by β-OHB. Notably, Kbhb of p53 was recently reported in the thymus (Liu et al., 2019), a typically less metabolic tissue, therefore indicating that Kbhb on specific proteins may be more nuanced.

Ultimately, it is the activated, CoA-bound form of β-OHB that serves as the substrate for Kbhb (Xie et al., 2016); however, the exact source of acylation-promoting acyl-CoAs for many PTMs including β-OHB is unclear. There are several possibilities for β-OHB-CoA. Acyl-CoA synthetase short chain 2 (ACSS2) is a nucleo-cytoplasmic enzyme that converts acetate to acetyl-CoA, supporting acetylation of histones (Mews et al., 2017; Sahar et al., 2014), as well as crotonate to crotonyl-CoA and histone crotonylation (Sabari et al., 2015). It is theorized that ACSS2 acts broadly to generate activated CoA forms of short-chain fatty acids and β-OHB is a candidate substrate (Sabari et al., 2017). Another possibility is that β-OHB-CoA is generated by flux through mitochondrial pathways as an intermediate metabolite (Ringel et al., 2018). Two forms of β-OHB-CoA are known to be generated through fatty acid β-oxidation; acetoacetyl-CoA is converted to L-β-OHB-CoA by mitochondrial 3-hydroxyacyl-CoA dehydrogenase (Reed and Ozand, 1980), and a yet-to-bedefined enzyme generates S-β-OHB-CoA from an acyl-CoA precursor (Robinson and Williamson, 1980). β-oxidation is ramped up under fasting, starvation, and KD and, once rates approach saturation, β-OHB-CoA concentration could rise substantially to out-compete other acyl-CoA species for reactivity with lysines or as a substrate for a yet-to-be-defined enzymatic writer. Our *in vitro* results demonstrate non-enzymatic Kbhb at high, yet physiologically relevant, concentrations of β-OHB-CoA (Wagner et al., 2017). Nevertheless, an enzyme-catalyzed mechanism is also likely for Kbhb deposition. The acetyltransferase p300 binds an array of acyl-CoAs (Kaczmarska et al., 2017), writes several other acyl PTMs (Huang et al., 2018a, 2018b; Sabari et al., 2015), and has been shown to facilitate histone Kbhb in a cell-free assay, and hence it is a candidate β-hydroxybutyrylating enzyme on non-histone proteins (Huang et al., 2021).

Similar to what is observed for other acylations (Wagner and Hirschey, 2014; Choudhary et al., 2009), macronutrient pathways were enriched for Kbhb including fatty acid β-oxidation, TCA cycle, glycolysis, amino acid metabolism, urea cycle, and ketogenesis. Several rate-limiting enzymes therein have more than 10 Kbhb sites: CPS1 (urea cycle), CS (TCA cycle), and HMGCS2 (ketogenesis). These metabolic networks are paramount for the fuel switching that must occur in the liver for a proper adaptation to ketogenic conditions, whereby fatty acid oxidation and amino acid catabolism fuel ketogenesis whereas gluconeogenesis maintains circulating glucose levels. Therefore, Kbhb appears to link cellular metabolism to the regulation of vital pathways at the global proteomic level. Intriguingly, all the enzymes participating in the production of β-OHB are modified by Kbhb. These include the rate-limiting HMGCS2 and the mitochondrial 3-hydroxyacyl-CoA dehydrogenase (HADH), which produces β-OHB-CoA (Reed and Ozand, 1980). Thus, we envision that Kbhb feeds back onto the pathways that generate its parent molecule as well as its activated CoA form.

Protein acetylation, which is also induced by fasting and a KD (Zhao et al., 2010; Roberts et al., 2017), occurs in parallel with Kbhb. When limiting analysis to mitochondria, ~75% of the proteins and sites of Kbhb overlap with Kac (Rardin et al., 2013b). Because our identification of Kbhb peptides by LC-MS/MS was performed in the presence of increased acetylation, it is possible that β-OHB-CoA competes with acetyl-CoA for these sites. Maximal ketogenic conditions might favor higher concentrations of β-OHB-CoA, because to a significant degree the fate of acetyl-CoA in the liver is β-OHB (Newman and Verdin, 2017). However, a limitation of our MS data is that the relative stoichiometry of Kbhb to Kac or unmodified lysine is not defined. The stoichiometry at specific lysine residues is crucial for the regulation of metabolic enzymes, as hypothesized for the competition between other acyllysine modifications such as acetylation, malonylation, and succinylation (Nishida et al., 2015).

Interestingly, the liver and kidney share one-carbon metabolism, a metabolic network that integrates several nutrients to fuel a variety of physiological processes, including nucleotide metabolism, amino acid homeostasis, lipid biosynthesis, redox balance, and methylation metabolism (Ducker and Rabinowitz, 2017). Several enzymes of the methionine cycle, a major arm of one-carbon metabolism, were β-hydroxybutyrylated in liver (Figure 3). The methionine cycle is of particular physiological importance because it regulates all cellular *trans*-methylation reactions by controlling the ratio of S-adenosylmethionine (SAM) and S-adenosylhomocysteine (SAH), and is therefore a critically controlled metabolic pathway. Notably, several enzymes identified in our (LC-)MS/MS analysis belong to this pathway. These include serine hydroxymethyltransferase (SHMT1), betaine-homocysteine methyltransferase (BHMT), glycine-*N*-methyltransferase (GNMT), and AHCY (Figure 4A). These enzymes function at several key steps of the cycle and together contribute to a tight control of SAM and SAH cellular levels (Girgis et al., 1998; Pajares and Pérez-Sala, 2006; Yeo and Wagner, 1992), suggesting that protein β-hydroxybutyrylation may play a role in methionine homeostasis under conditions of metabolic stress, such as prolonged fasting.

Many of the Kbhb sites identified in this study overlap with lysine residues important for enzymatic function, giving Kbhb the potential to modulate enzymatic activity. For example, CPS1, a rate-limiting enzyme in the urea cycle, is modified at 35 sites, including lysine residues previously linked to posttranslational control of its activity (Tan et al., 2014). This is the case for AHCY, whose β-hydroxybutyrylation decreases its enzymatic activity, demonstrating a functional relevance of the modification. In addition to the sites directly affecting activity, AHCY was modified at two additional sites in the N-terminal domain, which we hypothesize could affect protein-protein interactions or subcellular localization of AHCY (Grbeša et al., 2017). Future studies will help define the full array of functional consequences of Kbhb on target proteins and whether, as observed for AHCY, Kbhb targets other metabolic enzymes in a similar manner.

Global protein β-hydroxybutyrylation could play a significant role in metabolic adaptation in certain diseases. The ketogenic pathway has been linked to cancer (Kang et al., 2015; Poff et al., 2014); for example, a KD can elicit poorly understood anti-tumor effects (Weber et al., 2018). Because cancer cells often have impaired mitochondrial function, they are unable to utilize ketone bodies as an energy source (Maurer et al., 2011). This opens the possibility that, when held under a KD, cancer cells may accumulate β-OHB and consequently β-OHB-CoA and Kbhb. Cancer cells are highly dependent on methionine metabolism and several studies report AHCY as a possible therapeutic target (Belužić et al., 2018; Chayka et al., 2015). Moreover, in keeping with our results, variations in methionine metabolites have been linked to beneficial effects of KDs (Pissios et al., 2013), which presumably operate, at least in part, through protein Kbhb.

## STAR⋆METHODS

### RESOURCE AVAILABILITY

#### Lead contact

Further information and requests for resources and reagents should be directed to and will be fulfilled by the lead contact, Kevin Koronowski (kkoronow@hs.uci.edu)

#### Materials availability

This study did not generate new unique reagents

#### Data and code availability

- Proteomics data have been deposited at the ProteomeXchange Consortium via the PRIDE (Vizcaíno et al., 2016) partner repository with the dataset identifier ProteomeXchange: PXD016654 and are publicly available as of the date of publication. Accession numbers are listed in the Key resources table. Data are also available in Table S1.
- This paper does not report original code.
- Any additional information required to reanalyze the data reported in this paper is available from the lead contact upon request.

### EXPERIMENTAL MODEL AND SUBJECT DETAILS

#### Animals

Animal experiments were conducted in accordance with the guidelines of the Institution Animal Care and Use Committee (IACUC) at UC Irvine. Consideration was given to the ARRIVE guidelines during the development of the experimental design. Unless otherwise stated, adult male (8 to 16 weeks of age) C57BL/6J mice were group housed under a 12 hr light, 12 hr dark cycle at room temperature and fed *ab libitum* with normal chow (2020X Envigo, Teklad). Mice were randomly assigned to experimental groups. For fasting experiments, food was withdrawn at *Zeitgeber* time (ZT) 8, and tissue was harvested at ZT8 either 24 or 48 hr later. Tissues from mice fed control chow (4 weeks, TD.150345 Envigo, Teklad), ketogenic diet (4 weeks, TD.160153 Envigo, Teklad) or 10% w/w 1,3-butanediol diet (3 weeks, TD. 160257 Envigo, Teklad) were also harvested at ZT8. To induce type I diabetes mellitus (TIDM), mice were injected IP with 200 mg/kg streptozotocin (Caymen Chemical, 13104). Blood measurements and tissue harvest occurred 4 days after injections when the induction of TIDM was evident.

#### Cell lines

Mouse embryonic fibroblasts (MEF), human embryonic kidney (HEK) 293T and mouse hepatoma (Hepa1c1c) cells were cultured in DMEM (4.5 g l-^1^ glucose, Hyclone) supplemented with 10% FBS (GIBCO) and 100 units (penicillin), 100 μg (streptomycin) antibiotics (GIBCO). To generate stable expression of HA-AHCY in MEF cells, lentiviral HA-AHCY expression vector (VectorBuilder) was transfected into HEK293T cells together with psPAX2 and VSVG second-generation lentiviral packaging systems using BioT reagent (Bio-land Scientific LLC), according to manufacturer’s instructions. After 48h, lentiviral particles in the medium were collected, filtered and used to infect MEFs. 48h post transduction, the cells were subjected to hygromycin (Sigma) selection. Overexpression efficiency was verified by western blotting. (R)-(−)-3-hydroxybutyric acid sodium salt (Sigma 298360) was added to the culture media at the indicated concentrations. For dose response experiments, cells were treated for 24 hr. For time course experiments, treatment times were as indicated on figures and in legends. 293T were transfected with WT or mutant HA-AHCY plasmids using BioT reagent, according to manufacturer’s instructions.

### METHOD DETAILS

#### Immunoprecipitation

Whole cell lysates were prepared as described in Western blot. 500 μg of protein was incubated overnight at 4°C with primary antibody as indicated (anti-AHCY [MBL RN126PW], anti-HA [Millipore 05–904], anti-IgG Rb [Invitrogen 10500C], anti-acetyllysine [Cell Signaling 9441], Anti-3-hydroxybutyryllysine [PTM Biolabs 1201]. 20 μl of Protein G Dynabeads (Thermo Fisher Scientific) were added to the samples and incubated on a rocker at 4°C for 2 hr. Beads were then washed 4X with RIPA buffer (see Western blot). For western blot, bound proteins were eluted directly in SDS loading buffer. For AHCY activity, beads were added directly to the reaction mix. For immunodepletion experiments, 10 μg of antibody was added to 100 μg of protein lysate. For primary antibodies raised in rabbit, an anti-light chain, HRP-conjugated secondary antibody (Millipore MAB201P) was used to develop blots.

#### Subcellular fractionation

Mouse livers were homogenized with a motorized tissue grinder in STM buffer (Tris-HCl pH 7.4, 5 mM MgCl_2_, 250 mM Sucrose, 1 mM DTT, supplemented with 500 μM PMSF, Protease Inhibitor Cocktail [Roche, Basel, Switzerland], 10 mM nicotinamide and 330 nM TSA). Samples were then passed through a 100 μM filter, let on ice for 10 min and vortexed at max speed for 10 s. Nuclei were pelleted by centrifugation at 800 g for 10 min at 4°C and set aside. The same centrifugation was repeated on the supernatant to further remove nuclei. Next, mitochondria were pelleted by centrifugation at 11,000 g for 10 min at 4°C and set aside. Again, centrifugation was repeated on the supernatant to further remove mitochondria. The supernatant, which we took as the cytosolic fraction, was finally centrifuged at max speed for 10 min to remove any remaining nuclear or mitochondrial contamination. The nuclear pellet was next resuspended in CYTO buffer (10 mM HEPES-NaOH pH 8.0, 25 mM KCl, 650 μM spermidine, 1 mM EDTA, 1 mM EGTA, 340 mM sucrose, 1% NP40, 1 mM DTT, supplemented with 500 μM PMSF, Protease Inhibitor Cocktail, 10 mM nicotinamide and 330 nM TSA) and centrifuged at 800 g for 10 min at 4°C to clean the fraction. The final nuclear pellet was lysed in RIPA buffer (50 mM tris-HCl pH 8.0, 150 mM NaCl, 5 mM EDTA, 15 mM MgCl^2^ and 1% NP-40, supplemented with 500 μM PMSF, Protease Inhibitor Cocktail, 10 mM nicotinamide and 330 nM TSA). The Mitochondrial pellet was resuspended in STM buffer and centrifuged at 11,000 g for 10 min at 4°C to clean the fraction. The final mitochondrial pellet was lysed in RIPA buffer. Both nuclear and mitochondrial fractions were then sonicated at 60% amplitude, 5 s on 5 s off, for 20 s. Samples were then centrifuged at max speed for 10 min at 4°C to remove debris and membranes. From these fractions, samples were prepared for western blotting as described in Western blot.

#### Histone extraction

Liver tissue was homogenized with a motorized tissue grinder in extraction buffer (PBS, 0.5% Triton X-100 [v/v], supplemented with 500 μM PMSF, Protease Inhibitor Cocktail, 10 mM nicotinamide and 330 nM TSA) and lysed on ice for 10 min. Samples were centrifuged at 6,500 g for 10 min at 4°C to pellet nuclei, which were then washed in extraction buffer by repeating the previous centrifugation. Nuclei were resuspended in 0.2 M HCl overnight at 4°C. Following centrifugation at 6,500 g for 10 min at 4°C, the supernatant was mixed with a final concentration of 33% TCA to precipitate histones on ice for 30 min. Histones were pelleted by centrifugation at max speed for 10 min at 4°C. Pellets were then washed with ice cold 100% acetone and centrifugation was repeated for 2 washes. Final histone pellets were air-dried for 20 min at room temperature, dissolved in ddH_2_O and prepared with SDS loading buffer for western blot as described. The amount of histones per sample was compared by ponceau staining and normalized accordingly for subsequent blotting.

#### *β*-hydroxybutyrate and glucose measurements

Blood β-hydroxybutyrate levels were measuring with a Nova Max Plus^™^ β-ketone meter (Nova Biomedical). Blood glucose was measured with a Contour blood glucose monitor (Bayer). Readings were acquired by a ~2 mm tail cut.

#### AHCY activity assay

To measure the enzymatic activity of AHCY from total lysates, samples were incubated in homogenization buffer, provided in the Adenosylhomocysteinase (AHCY) Activity Fluorometric Assay Kit (Biovision, K807–100), and let on ice for 15 min at 4°C, followed by centrifugation at 14,000 rpm for 10 min at 4°C. Pellets were then resuspended in AHCY assay buffer. Activity of AHCY from total lysates and immunoprecipitated AHCY was measured according to the manufacturer’s instructions. The assay was performed in 96-well white plates and fluorescence was measured in kinetic mode for 30–45 min at 37°C using a Varioskan LUX multimode microplate reader (Thermo Fisher Scientific).

#### AHCY mutagenesis

HA-AHCY was purchased from VectorBuilder and mutations were introduced by PCR- based mutagenesis using Q5 Site-Directed Mutagenesis Kit (NEB) according to manufacturer’s instructions. Primers were as follows: K405R FOR 5′-acctgggcaagctgaatgtgagg ctgaccaagc-3′, REV 5′-gcttggtcagcctcacattcagcttgcccaggt-3′; K389R FOR 5′-gggttcacttcctgcctaagaggctggatgagg-3′, REV 5′-cctc atccagcctcttaggcaggaagtgaaccc-3′; K188R FOR 5′-attctgtcaccaagagcaggtttgacaacctctatgg-3′, REV 5′-ccatagaggttgtcaaacctg ctcttggtgacagaat-3′; K204R FOR 5′-gatggcatcagacgggccaca-3′ REV 5′-tatgagggactcccggca-3′. Mutagenesis was confirmed by sequencing.

#### AHCY protein expression and purification

Mouse AHCY residues 1–432 were cloned into the pHIS-parallel vector, which adds a His_6_ tag at the N terminus, using the BamHI and Sal1 sites. His_6_-AHCY was expressed in the BL21 (DE3) strain of *E. coli*. Cells were grown at 37°C with shaking until they reached an OD_600_ of 0.8, whereupon His_6_-AHCY expression was induced with 0.5 mM isopropyl-β-D-thiogalactopyranoside (IPTG) and then grown for an additional 16 h at 18°C. Cells were harvested by centrifugation at 4000 × rpm and resuspended in lysis buffer (50 mM Tris pH 8.0, 500 mM NaCl and 20 mM imidazole). Cells were lysed using a microfluidizer followed by sonication on ice (15 s on, 45 s off for four pulses at 40% amplitude with a 1/4” microtip). His_6_-AHCY was isolated by Ni^2+^-nitrilotriacetic acid (Ni-NTA) affinity chromatography (QIAGEN) using standard procedures and eluted with 50 mM Tris buffer pH 8.0, 500 mM NaCl and 300 mM imidazole. The His_6_ tag was cleaved with His_6_-TEV protease overnight at 4°C. The Ni-NTA eluate was then diluted with lysis buffer to lower the concentration of imidazole to 40 mM and subsequent Ni-NTA affinity chromatography was performed to remove His_6_ tag and His_6_-Tev.

Preparation of NAD+-bound holoenzyme was performed as previously described (Manszewski et al., 2017). Briefly, 5 mL of apo AHCY (at a concentration of 12 mg/mL) was mixed with 10 mL of saturated ammonium sulfate solution at pH 3.3 and incubated on ice for 10 minutes. The protein was then pelleted by centrifugation at 10,000 × rpm for 15 minutes at 4°C. The precipitate was dissolved in 5 mL of lysis buffer and mixed with 10 mL of saturated ammonium sulfate solution at pH 3.3, incubated and centrifuged again. Supernatant was discarded and the pellet was again diluted in lysis buffer and mixed with 10 mL of saturated ammonium sulfate solution at pH 7.0. The mixture was centrifuged again and the resulting pellet resuspended in lysis buffer. Protein was incubated overnight at 4°C with 12-fold excess of NAD+. The preparation was further purified by Superdex 200 gel filtration chromatography (GE Health-care) into 20 mM Tris pH 8.0, 50 mM NaCl. The protein was aliquoted, frozen in liquid nitrogen, and stored at −80°C for storage.

#### AHCY *in vitro* acylation

A cell free assay to induce β-hydroxybutyrylation of AHCY was performed as described previously with adjustments (Kaczmarska et al., 2017). Reactions were set up in reaction buffer (25 mM Tris-HCl pH 8.0, 100 mM NaCl, 1 mM DTT, 100 μM EDTA, 10% glycerol, supplemented with Protease Inhibitor Cocktail, 10 mM nicotinamide and 100 ng/ml TSA), with 2 μg AHCY and 100 μM, 1 mM or 10 mM DL-β-hydroxybutyryl-CoA lithium salt (Sigma H0261), for 2 hr in a 37°C water bath. Reactions were either stopped with addition of SDS loading buffer for western blot or loaded directly into AHCY activity assay wells for measurement.

#### Western blot

Whole cell lysates were prepared by homogenizing cells or tissue with a motorized tissue grinder in RIPA buffer (see Subcellular fractionation for recipe). Samples were lysed on ice for 20 min, vortexed briefly and sonicated at 60% amplitude, 5 s on 5 s off, for 4 cycles. Membranes and debris were removed by centrifugation at max speed for 10 min at 4°C. Protein concentration was measured via the Braford method with Protein Assay Dye from BioRad (Hercules, CA). Ten to 50 μg protein was loaded into a 12% SDS-PAGE gel. Following transferal to a nitrocellulose membrane (Thermo Fisher Scientific, Waltham, MA), we blocked with 5% milk in TBST (0.1% Tween-20, Tris Buffered Saline) for 2 hr at room temperature. Primary antibodies (Anti-3-hydroxybutyryllysine [PTM Biolabs 1201], anti-p84 [GeneTex GTX70220], anti-α-Tubulin [Sigma T5168], anti-COXIV [Cell Signaling 11967S], anti-acetyllysine [Cell Signaling 9441], anti-AHCY [Abcam ab134966], anti-HA [Millipore 05–904]) were diluted in 5% milk TBST and incubated with membranes overnight at 4°C. Membranes were then incubated with HRP-conjugated secondary antibodies (Mouse IgG-HRP conjugate [EMD Millipore AP160P], Rabbit IgG-HRP linked [EMD Millipore 12–348]) for 1 hr at room temperature. Blots were developed with Immobilon Western Chemiluminescent HRP Substrate (Millipore, Burlington, MA) and HyBlot CL autoradiography film (Denville Scientific, Holliston, MA).

#### Proteomics

To qualitatively identify Kbhb peptides, liver from a starved (48 hr fasted) mouse was cut into small pieces, washed with cold PBS and homogenized on ice in lysis buffer (8 M urea, 2 mM EDTA, 3 μM TSA, 50 mM NAM, 5 mM DTT and 1% Protease Inhibitor Cocktail III). The remaining debris was removed by centrifugation at 18,000 × g at 4°C for 3 min. Proteins in the cell lysate were reduced with 10 mM DTT for 1 hr at 37°C, alkylated with 20 mM iodoacetamide for 45 min at room temperature in darkness, and the excess iodoacetamide was blocked by 20 mM cysteine. Then the protein sample was diluted by adding 100 mM NH_4_HCO_3_ to reduce the urea concentration to 1 M. Trypsin was added at 1:50 trypsin-to-protein mass ratio for the first digestion overnight and 1:100 trypsin-to-protein mass ratio for a second 4 hr digestion. Finally, 18 mg of protein was digested for subsequent experiments. Peptides were fractionated into 23 fractions by high pH reverse-phase HPLC using an Agilent 300 Extend C18 column (5 μm particles, 4.6 mm ID, 250 mm length). Briefly, peptides were first separated using a gradient of 2%–60% acetonitrile in 10 mM ammonium bicarbonate at pH 10 over 90 min into 90 fractions. The peptides were then combined into 23 fractions and dried by vacuum centrifugation, then each fraction was subjected to enrichment individually. To enrich Kbhb peptides, total peptides dissolved in NETN buffer (100 mM NaCl, 1 mM EDTA, 50 mM Tris-HCl, 0.5% NP-40, pH 8.0) were incubated with pre-washed pan anti-Kbhb beads (30 uL, PTM Biolabs Inc., Chicago, IL) at 4°C overnight with gentle shaking. The beads were washed four times with NETN buffer and twice with ddH_2_O. The bound peptides were eluted from the beads with 0.1% trifluoroacetic acid, and the eluted fractions were combined and vacuum-dried. Next, each enriched fraction was subjected to LC-MS/MS separately. Dynamics of total peptides were not assessed. Samples were dissolved in 0.1% formic acid and loaded onto a home-made capillary column (10 cm length with 75 um inner diameter) packed with Reprosil 100 C18 resin (3 μm particle size, Dr. Maisch GmbH, Ammerbuch, Germany). Peptide separation was performed with a gradient of 5%–80% HPLC buffer B (0.1% formic acid in 90% acetonitrile, v/v) in buffer A (0.1% formic acid in water, v/v) at a flow rate of 300 nl/min over 60 min on an Eksigent UPLC system. The samples were analyzed by Orbitrap Velos Mass Spectrometers (ThermoFisher Scientific). A data-dependent procedure that alternated between one full mass scan followed by the top 20 most intense precursor ions was applied with 90 s dynamic exclusion. Intact peptides were detected with a resolution of 30,000 at 400 m/z, and ion fragments were detected at 35%.

#### Proteomic bioinformatic analysis

Database searching was performed with MaxQuant 1.3.0.5 (Cox and Mann, 2008) against the mouse proteome downloaded from UniProt. Trypsin was specified as cleavage enzyme allowing a maximum of 2 missing cleavages. Cysteine carbamidomethylation was set as a fixed modification. Methionine oxidation, N-terminal acetylation of proteins, and lysine β-hydroxybutyrylation were set as variable modifications. The false discovery rate (FDR) was set to 1% for peptide, protein and modification sites. Peptides identified from reverse or contaminant protein sequences, peptides with score below 40 or site localization probability below 0.75 were removed.The flanking sequence motif of lysine β-hydroxybutyrylation substrates were tested against all mouse background sequences with iceLogo (Colaert et al., 2009) using a p value of 0.05.

#### Metabolite extraction

To measure SAH, frozen tissue samples were ground at liquid nitrogen temperature with a Cryomill (Retsch, Newtown, PA). The resulting tissue powder (~20 mg) was weighed and then extracted by adding −20°C 40:40:20 (methanol:acetonitrile:water) mixture, vortexed, and centrifuged at 16,000 × g for 10 min at 4°C. The volume of the extraction solution (μL) was 40x the weight of tissue (mg) to make an extract of 25 mg tissue per mL solvent. The supernatant (40 μL) was collected for LC-MS analysis.

#### Measurement of metabolites

A quadrupole-orbitrap mass spectrometer (Q Exactive, Thermo Fisher Scientific, San Jose, CA) operating in a positive ion mode was coupled to vanquish UHPLC system (ThermoFisher Scientific, San Jose, CA) with electrospray ionization and used to scan from m/z 70 to 1000 at 1 Hz and 75,000 resolution. The LC separation was achieved on a XBridge BEH Amide column (2.1 mm × 150 mm, 2.5 μm particle size, 130 Å pore size; Waters, Milford, MA) using a gradient of solvent A (95:5 water: acetonitrile with 20 mM ammonium acetate and 20 mM ammonium hydroxide, pH 9.45) and solvent B (acetonitrile). Flow rate was 150 μL/min. The LC gradient was: 0 min, 85% B; 2 min, 85% B; 3 min, 80% B; 5 min, 80% B; 6 min, 75% B; 7 min, 75% B; 8 min, 70% B; 9 min, 70% B; 10 min, 50% B; 12 min, 50% B; 13 min, 25% B; 16 min, 25% B; 18 min, 0% B; 23 min, 0% B; 24 min, 85% B; 30 min, 85% B. Autosampler temperature was 5°C, and injection volume was 3 μL. Data were analyzed using the MAVEN software.

#### Homocysteine measurement

Homocysteine was measured in serum using the Homocysteine ELISA Kit (BioVision, E4543-100) according to the manufacturer’s instructions.

### QUANTIFICATION AND STATISTICAL ANALYSIS

Statistical test, significance threshold and number of biological replicates information for each experiment is in the figure legends or corresponding methods section. Unless otherwise noted, all data are presented as mean ± SEM. Mice were randomized into groups using a random number generator. Sample size was determined by referencing standards in the literature as well as calculating statistical power with G*Power 3.1.9.7 software. Statistical tests were carried out, and graphs were made, in GraphPad Prism 6.0 software. All data were analyzed with parametric tests assuming Gaussian distribution.

## Supplementary Material

1

2

## Figures and Tables

**Table:** KEY RESOURCES TABLE

REAGENT or RESOURCE	SOURCE	IDENTIFIER
Antibodies		
anti-AHCY	MBL	RN126PW
anti-HA	Millipore	05–904; RRID:AB_417380
anti-IgG Rb	Invitrogen	10500C; RRID:AB_2532981
anti-acetyllysine	Cell Signaling	9441; RRID:AB_331805
Anti-3-hydroxybutyryllysine	PTM Biolabs	1201
anti-light chain, HRP-conjugated	Millipore	MAB201P; RRID:AB_827270
anti-p84	GeneTex	GTX70220; RRID:AB_372637
anti-α-Tubulin	Sigma	T5168; RRID:AB_477579
anti-COXIV	Cell Signaling	11967S; RRID:AB_2797784
anti-AHCY	Abcam	ab134966
Mouse IgG-HRP conjugate	Millipore	AP160P; RRID:AB_92531
Rabbit IgG-HRP linked	Millipore	12–348; RRID:AB_390191
Chemicals, peptides, and recombinant proteins		
Streptozotocin	Caymen Chemical	13104
cOmplete Protease Inhibitor Cocktail	Roche	11697498001
DL-β-hydroxybutyryl-CoA lithium salt	Sigma	H0261
BioT transfection reagent	Bioland Scientific	B01–00
Critical commercial assays		
Adenosylhomocysteinase (AHCY) Activity Fluorometric Assay Kit	Biovision	K807–100
Q5 Site-Directed Mutagenesis Kit	New England Biolabs	E0554S
(R)-(-)-3-hydroxybutyric acid sodium salt	Sigma	298360
Homocysteine ELISA Kit	BioVision	E4543–100
Deposited data		
Identified Kbhb peptides (see also Table S1)	ProteomeXchange	PXD016654
Experimental models: Cell lines		
Mouse embryonic fibroblasts (MEF)	NA	NA
Human embryonic kidney (HEK) 293T	NA	NA
Mouse hepatoma (Hepa1c1c)		NA
Experimental models: Organisms/strains		
C57BL/6J mouse	The Jackson Laboratory	000664
Other		
Protein G Dynabeads	Thermo Fisher Scientific	10003D
Normal chow diet (animal house)	Envigo, Teklad	2020X
Control chow (experimental)	Envigo, Teklad	TD.150345
Ketogenic diet	Envigo, Teklad	TD.160153
1,3-butanediol diet	Envigo, Teklad	TD. 160257
anti-Kbhb beads	PTM Biolabs	1204

## Highlights

- Lysine β-hydroxybutyrylation (Kbhb) modifies non-histone proteins in liver and kidney
- Starvation, ketogenic diet, and pharmacologically induced diabetes evoke Kbhb
- LC-MS/MS identifies 891 Kbhb-modified peptides enriched for metabolic pathways
- Kbhb inhibits the rate-limiting methionine cycle enzyme AHCY
