# Supplementary material for: Ketogenesis impact on liver metabolism revealed by proteomics of lysine β-hydroxybutyrylation
Source: Cell Rep. Author manuscript; Available in PMC 2021 Aug 18. (PMC8372761; doi:10.1016/j.celrep.2021.109487)
Supplement: 1 [file NIHMS1730377-supplement-1.pdf]

Cell Reports, Volume *36*

Supplemental information

Ketogenesis impact on liver metabolism revealed by proteomics of lysine β-hydroxybutyrylation

Kevin B. Koronowski, Carolina M. Greco, He Huang, Jin-Kwang Kim, Jennifer L. Fribourgh, Priya Crosby, Lavina Mathur, Xuelian Ren, Carrie L. Partch, Cholsoon Jang, Feng Qiao, Yingming Zhao, and Paolo Sassone-Corsi

SUPPLEMENTARY FIGURES AND LEGENDS

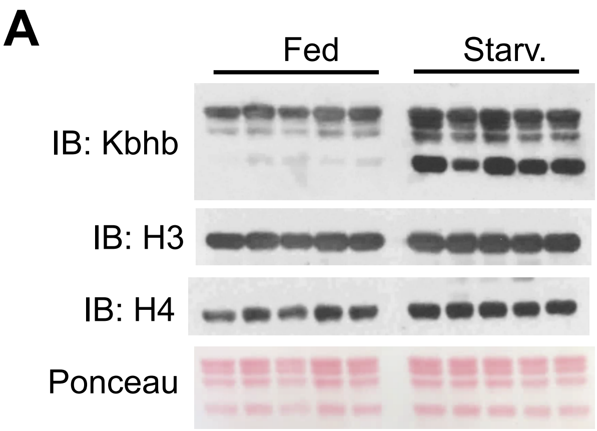

Figure S1. Induction of Kbhb on liver histones. Related to Figure 1. (A) Western blot of histone extracts from fed and starved (48 hr fast) livers.

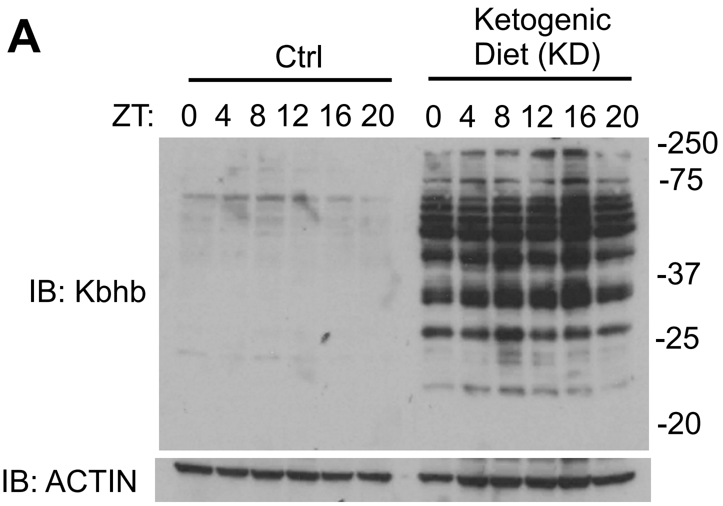

Figure S2. Induction of Kbhb by ketogenic diet over the diurnal cycle. Related to Figure 2. (A) Whole cell lysates of livers harvested at 6 different time points over the circadian cycle. ZT0=lights on, ZT12=lights off.

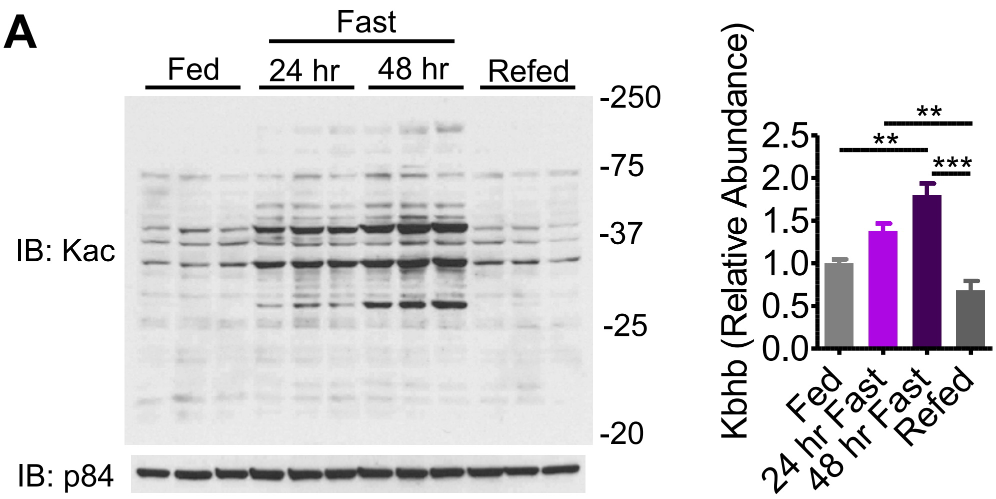

Figure S3. Induction of Kac in fasted liver. Related to Figure 3. (A) Whole cell lysates from fed and starv. (48 hr fast) liver. n=3 replicates are quantified to the right, unpaired Student's t-test, $** = p<0.01$, $*** = p<0.001$. Data are represented as mean ± SEM.

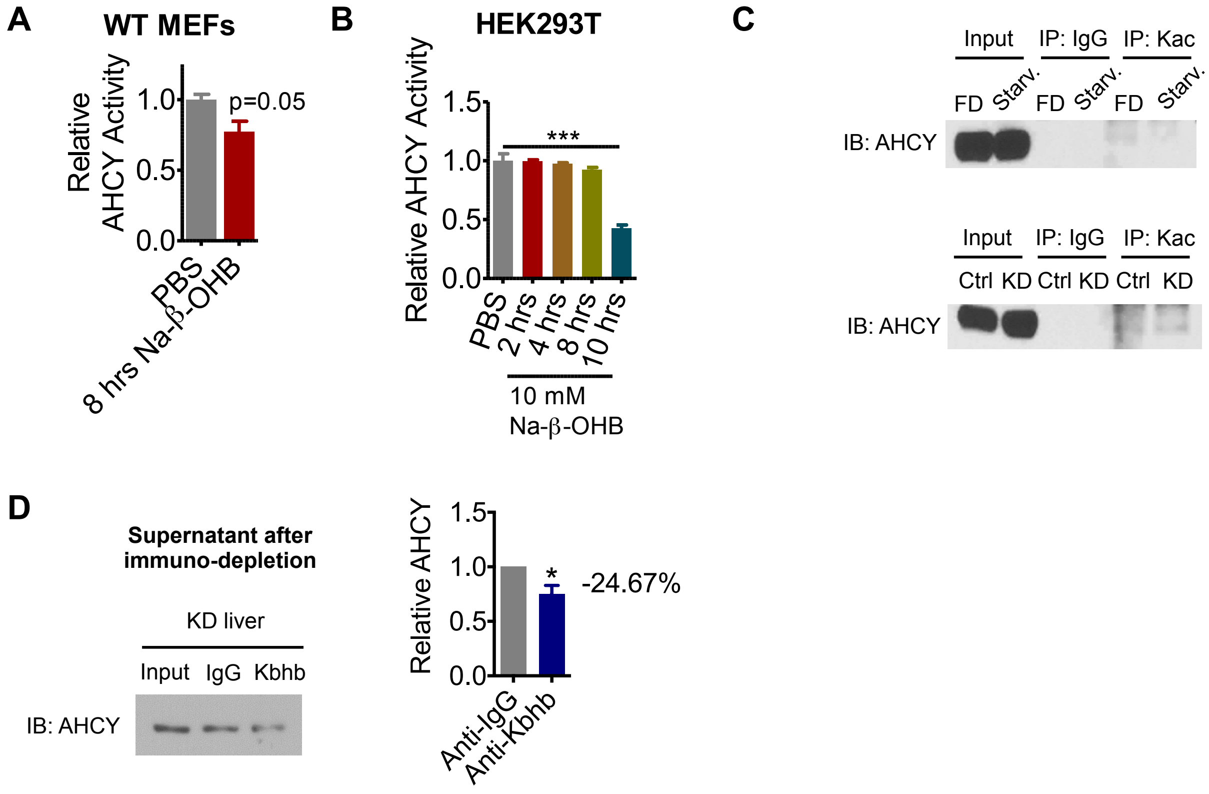

Figure S4. Effects of β-hydroxybutyrate on AHCY in vitro and in vivo. Related to Figure 4. (A-B) AHCY activity measured in whole cell lysates of WT MEF (A) and HEK293T (B) cells. (A) Unpaired Student's t-test, n=3. (B) One-way ANOVA, ***=p<0.001, n=3-6. (C) Immunoprecipitation with Pan-Anti-K-acetyl antibody (Kac) from liver whole cell lysates. FD – fed; Starv. – 48 hr fast; Ctrl – control diet; KD – ketogenic diet. (D) Left – peptides were depleted from ketogenic diet (KD) liver whole cell lysates with either rabbit IgG or Pan-Anti-K-bhb antibodies. The resulting depleted supernatants were blotted for AHCY. Right – quantification of n=3 replicates, where values are normalized to IgG. Unpaired Student's t-test, *=p<0.05. Data are represented as mean ± SEM.

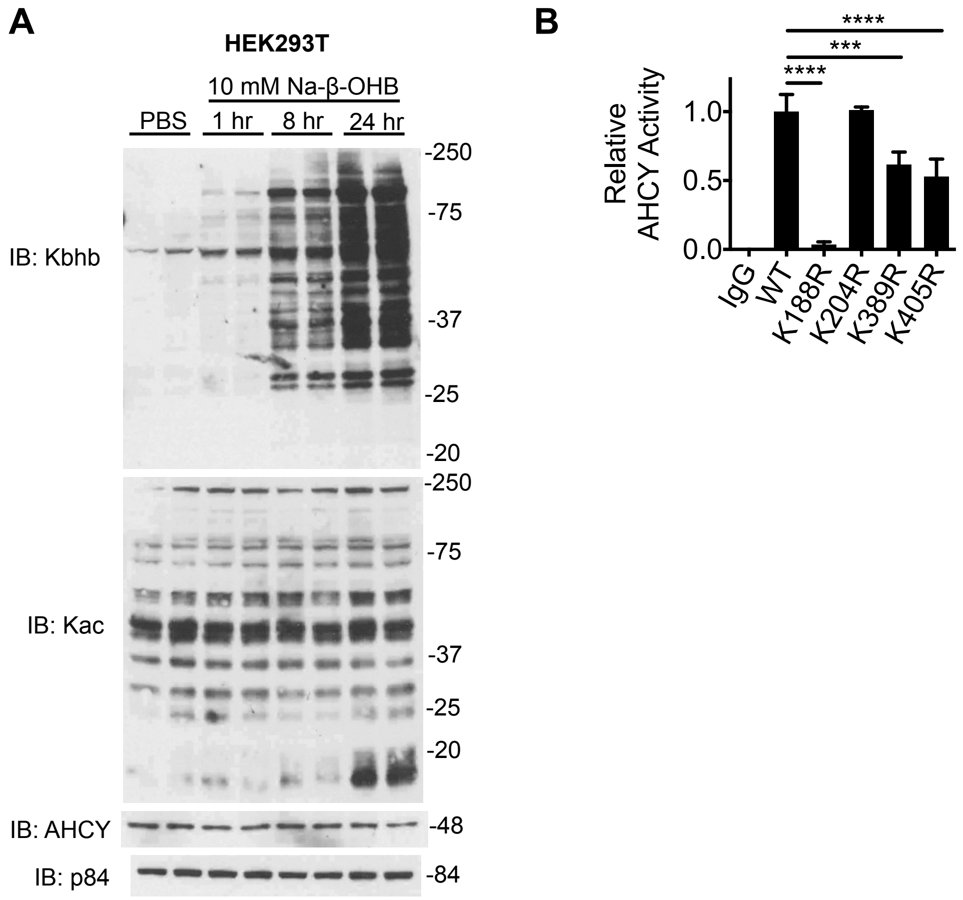

Figure S5. Expression of AHCY mutants in HEK293T cells. Related to Figure 5. (A) Time course of Kbhb induction in HEK293T cells following sodium-β-hydroxybutyrate (Na-β-OHB) treatment. (B) HEK293T cells were transfected with WT or the indicated lysine mutant plasmid, treated for 8-10 hr with PBS, then transfected AHCY was immunoprecipitated by its HA tag and assayed for enzymatic activity (as in Figure 5B). The effect of each mutation alone on AHCY activity can be appreciated. Values are normalized to WT. One-way ANOVA, Bonferroni posthoc test, ***=p<0.001, ****=p<0.0001 WT – n=6, mutants – n=3. Data are represented as mean ± SEM.
